# Correcting hyponatraemia is associated with improved survival in hyponatraemic metastatic cancer patients

**DOI:** 10.1093/ckj/sfaf023

**Published:** 2025-01-24

**Authors:** Kenneth Ward, Valda D Page, Juhee Song, Jaya Sheela Amaram-Davila, Omar Mamlouk, Ala Abudayyeh

**Affiliations:** Section of Nephrology, Division of Internal Medicine, University of Texas MD Anderson Cancer Center, Houston, TX, USA; Section of Nephrology, Division of Internal Medicine, University of Texas MD Anderson Cancer Center, Houston, TX, USA; Department of Biostatistics, University of Texas MD Anderson Cancer Center, Houston, TX, USA; Department of Palliative, Rehabilitation and Integrative Medicine, University of Texas MD Anderson Cancer Center, Houston, TX, USA; Section of Nephrology, Division of Internal Medicine, University of Texas MD Anderson Cancer Center, Houston, TX, USA; Section of Nephrology, Division of Internal Medicine, University of Texas MD Anderson Cancer Center, Houston, TX, USA

**Keywords:** hospice, hyponatraemia, stage IV cancer, survival

## Abstract

**Background:**

Hyponatraemia in cancer patients admitted to the hospital is associated with longer stays, higher costs and increased mortality. We examined the impact of hyponatraemia correction on survival in hospitalized patients with advanced cancer.

**Methods:**

We reviewed records of patients with solid tumours who were hospitalized between January 2018 and December 2022 with serum sodium ≤125 mEq/l at admission. Cox regression analysis examined associations of demographic and clinical characteristics, including sodium levels at admission and discharge, with overall survival.

**Results:**

Among 1100 patients, median sodium levels were 122 mEq/l at admission and 132 mEq/l at discharge. A total of 165 patients (15%) died during hospitalization and 414 of 688 discharged home (60.2%) died within 5 years. Multivariable analysis showed that among patients discharged alive, a decrease in sodium from admission to discharge (*P* = .0081), sodium ≤125 mEq/l at discharge [hazard ratio (HR) 1.42; *P* = .0382], albumin <3.5 g/dl at admission (HR 1.48; *P* < .0001), metastatic stage (HR 1.37; *P* = .0004), emergency admission (HR 1.20; *P* = .0390), discharge to hospice (HR 2.57; *P* < .0001), lung cancers (HR 1.51; *P* = .0044) and metastatic disease (HR 1.37; *P* = .0004) were associated with poorer overall survival. Sodium level at admission was not a significant predictor of overall survival from hospital admission. In patients with metastatic disease, an increase in sodium from admission to discharge was associated with improved overall survival from hospital discharge.

**Conclusions:**

Correcting hyponatraemia in hospitalized patients with metastatic cancer increases overall survival, but metastatic cancer in itself is also associated with poor survival. This highlights the importance of early palliative care involvement in patients with advanced cancer.

KEY LEARNING POINTS **What was known:**

- Hyponatraemia in hospitalized cancer patients is linked to longer hospital stays, higher healthcare costs and increased mortality.
- Correction of hyponatraemia in metastatic cancer patients was hypothesized to improve survival, but specific outcomes remained unclear, prompting further investigation.

**This study adds:**

- Sodium level improvement from admission to discharge is significantly associated with increased survival in metastatic cancer patients.
- Correcting hyponatraemia is essential for improving outcomes, emphasizing the need for prompt, targeted management in advanced cancer care.

**Potential impact:**

- This study supports integrating nephrology and palliative care to address hyponatraemia early, potentially improving survival outcomes and improving sodium management in cancer patients.

## INTRODUCTION

Hyponatraemia is the most common electrolyte abnormality, occurring in up to 30% of hospitalized patients, and has been associated with increased morbidity and mortality [1–3]. Hyponatraemia is typically associated with cardiac, liver and lung diseases, as well as the use of certain drugs and syndrome of inappropriate antidiuretic hormone. Hyponatraemia is defined as a serum sodium level of <135 mEq/l and further classified as mild (130–134 mEq/l), moderate (125–129 mEq/l) or severe (<125 mEq/l) [4, 5]. Hyponatraemia of 120–124 mEq/l in hospitalized patients was shown to be associated with a mortality rate of 11.2% [6].

In cancer patients, reported rates of hyponatraemia range from 1% to 76%, depending on the malignancy and definition of hyponatraemia [7, 8]. Hyponatraemia can delay the care of cancer patients, such as surgeries, and has been associated with increased morbidity and mortality. According to the World Health Organization, cancer is the second leading cause of death globally, responsible for 8.8 million deaths in 2015 [9]. Given this high rate of mortality, it has become imperative to reduce further complications such as hyponatraemia. In a cancer patient, causes of hyponatraemia are typically related to the cancer, such as increased antidiuretic hormone levels induced by tumour burden, especially with liver involvement (hepatorenal-like physiology), or by tumour secretions, pain or nausea. Cancer patients may also have a loss of sodium or potassium with secondary water retention (hypovolaemic) related to vomiting or diarrhoea after chemotherapy. Patients with advanced cancer also have cachexia syndrome, affecting their oral intake [10–12].

In several studies, hyponatraemia has been associated with poor survival and longer hospital stays in cancer patients [11–14]. There has been increased interest in evaluating the impact of hyponatraemia on survival in patients with metastatic solid tumours, as well as whether correction of sodium levels has a positive impact. Additionally, there is a growing consensus that a more guided, patient-centred approach should be taken at the end of life. Although hyponatraemia was associated with poorer survival in patients with advanced cancer in a few studies, the association does not imply causality, because hyponatraemia may also be a marker of progressive cancer [7, 8, 15–20].

In the current study, we analysed the impact of improved sodium levels on survival outcomes in patients with metastatic solid cancer and hyponatraemia admitted to a comprehensive cancer centre compared with outcomes in patients with non-metastatic cancer.

## MATERIALS AND METHODS

### Study design and patient population

This retrospective study was approved by the institutional review board at the University of Texas MD Anderson Cancer Center in accordance with the principles of the Declaration of Helsinki. We reviewed the charts of all patients with solid cancers who were admitted to MD Anderson with sodium levels ≤125 mEq/l between 1 January 2018 and 31 December 2022 and later had a nephrology consultation. Data were collected using SAP BusinessObjects BI Platform 4.2 (version 14.2.9.4473; SAP SE, Walldorf, Germany), which was linked to the institution's electronic medical records system, Epic (Epic Systems, Verona, WI, USA) for data extraction. We collected data related to patient demographics (age, sex and race/ethnicity), type of cancer, length of hospital stay, cost of hospital stay, admission unit/location [including length of intensive care unit (ICU) stay, if applicable], number of readmissions, discharge disposition, vital status at discharge and serum sodium values on the day of admission and discharge were extracted directly from Epic. Increased sodium or a change in sodium was defined as any 1 mEq/l increase in sodium levels from hospital admission to discharge. A total of 1221 records were extracted that met the study criteria. We excluded 121 additional admission records for patients with multiple admission records, ensuring only one admission record per patient was included in the analysis. The number of readmissions was counted for each patient and compared between metastatic and non-metastatic patients. The final study population was 1100 unique patients with sodium levels ≤125 mEq/l at admission.

### Statistical analysis

Patient demographic and admission characteristics were summarized using descriptive statistics according to disease stage (metastatic or non-metastatic). The metastatic and non-metastatic groups were compared by two-sample *t*-test or Wilcoxon rank-sum test for continuous variables and chi-squared test or Fisher’s exact test for categorical variables. The main endpoints of the study were long-term survival after hospital discharge, hospital survival from admission to discharge and overall survival after hospital admission.

Survival analysis was performed using univariate and multivariable Cox regression analysis (stepwise selection was used for model selection as appropriate) to assess long-term survival for the entire population and specifically for patients with metastatic disease. The analysis encompassed long-term survival after hospital discharge for all patients, excluding those discharged to hospice, as well as hospital survival (with survival times censored at discharge for those still alive) and overall survival time from hospital admission to last follow-up. Kaplan–Meier analysis was used to estimate survival probabilities and the logrank test was used to compare survival between groups. *P*-values <.05 indicated statistical significance. SAS version 9.4 (SAS Institute, Cary, NC, USA) was used for data analysis.

## RESULTS

### Patient characteristics

Of the 1100 patients in our analysis, 644 (58.5%) had metastatic cancer, and the median sodium level at admission for the entire cohort was 122 mEq/l [interquartile range (IQR) 119–124 mEq/l]. The median age was 64 years (IQR 55–71) and the median length of admission was 6 days (IQR 4–10). The median cost of the hospital admission was US$58 256 (IQR 34 236–101 976). The most common cancers were gastrointestinal [*n* = 408 (37.1%)], genitourinary [*n* = 142 (12.9%)] and lung [*n* = 135 (12.3%) (Table 1). Also, 336 patients (30.5%) had an ICU admission during their hospitalization. Compared with patients who did not have metastatic disease, those with metastatic cancer had a significantly higher proportion of gastrointestinal cancers (43.0% compared with 28.7%) and a significantly lower proportion of lung cancers (6.4% compared with 20.6%) (Table 1).

**Table 1:** Patient characteristics according to presence of metastatic disease.

Variables	Non-metastatic (*n* = 456)	Metastatic (*n* = 644)	*P-*value
Age at admission (years), median (IQR)	65 (57–72)	63 (54–71)	.0018
Sodium level at admission (mEq/l), median (IQR)	122 (119–124)	122 (119–124)	.4881
Albumin at admission (g/dl), median (IQR)	3.6 (3.1–4.1)	3.3 (2.8–3.8)	<.0001
Total charge for hospital stay (US$), median (IQR)	54 049.57 (32 261.43–99 971.71)	60 173.33 (36 650.77–104 765.12)	.0677
Hospital stay (days), median (IQR)	5.96 (3.88–10.13)	6.08 (3.75–10.44)	.9415
Sex, *n* (%)			.7719
Female	219 (48)	315 (48.9)	
Male	237 (52)	329 (51.1)	
Race, *n* (%)			.5108
White	335 (73.5)	451 (70.0)	
Black	32 (7.0)	50 (7.8)	
Asian	43 (9.4)	66 (10.2)	
Other	45 (9.9)	71 (11.0)	
Unknown	1 (0.2)	6 (0.9)	
Ethnicity			.7490
Hispanic/Latino	77 (16.9)	119 (18.5)	
Not Hispanic/Latino	369 (80.9)	513 (79.7)	
Unknown	10 (2.2)	12 (1.9)	
Admission type			.4239
Non-emergency	162 (35.5)	244 (37.9)	
Emergency	294 (64.5)	400 (62.1)	
Admission location			.4712
Floor	363 (79.6)	501 (77.8)	
Intensive care unit	93 (20.4)	143 (22.2)	
Insurance coverage			.0002
Commercial	230 (50.4)	407 (63.2)	
Government	16 (3.5)	15 (2.3)	
Medicaid/Medicare	183 (40.1)	183 (28.4)	
Self-pay	27 (5.9)	39 (6.1)	
Admitted with hyponatremia			.6332
No	127 (27.9)	171 (26.6)	
Yes	329 (72.1)	473 (73.4)	
Intensive care unit admission			.5689
Yes	135 (29.6)	201 (31.2)	
No	321 (70.4)	443 (68.8)	
Cancer type			<.0001
Brain	10 (2.2)	7 (1.1)	
Breast	31 (6.8)	48 (7.5)	
Gastrointestinal	131 (28.7)	277 (43.0)	
Genitourinary	59 (12.9)	83 (12.9)	
Gynaecologic	39 (8.6)	55 (8.5)	
Head and neck	45 (9.9)	39 (6.1)	
Lung	94 (20.6)	41 (6.4)	
Sarcoma	10 (2.2)	25 (3.9)	
Skin	37 (8.1)	69 (10.7)	
Discharge location			<.0001
Home	322 (70.6)	366 (56.8)	
Hospice	42 (9.2)	155 (24.1)	
Death	65 (14.3)	100 (15.5)	
Another care facility	27 (5.9)	23 (3.6)	
Comorbidity, *n* (%)			
CKD	89 (19.5)	99 (15.4)	.0720
Cirrhosis	27 (5.9)	64 (9.9)	.0172
Diabetes	6 (1.3)	4 (0.6)	.2317
Heart failure	35 (7.7)	40 (6.2)	.3425
Hypertension	48 (10.5)	56 (8.7)	.3066

The median sodium level at admission was 122 mEq/l (IQR 119–124) for both those with and those without metastatic cancer and thus did not significantly differ between groups (Table 1). However, median sodium levels at discharge were higher for patients without metastatic disease [132.5 mEq/l (IQR 130–136) compared with 132 mEq/L (IQR 128–135) for those with metastatic disease; *P* = .0003]. Accordingly, the median change in sodium levels at discharge was higher in patients without metastatic disease [11 mEq/l (IQR 7–15) compared with 10 mEq/L (IQR 6–14) for those with metastatic disease; *P* = .0027].

Patients with metastatic disease incurred a higher median total charge associated with their admission [US$60 173 (IQR 36 652–104 765) compared with US$54 050 (IQR 32 261–99 972) for those without metastatic disease; *P* = .0677) (Table 1).

There were more readmissions within the last 6 months in the metastatic group than in the non-metastatic group (*P* = .0376). In the non-metastatic group, 40% had readmissions, compared with 48% in the metastatic group. In addition, 7% of patients in the non-metastatic group had three or more readmissions, compared with 10% in the metastatic group.

### Survival analysis

In the overall population of 1100 patients, hospital deaths were reported for 165 (15.0%). Among the 935 discharged alive during the study period (1 January 2018–31 December 2022), 586 (62.7%) died when evaluated at the last follow-up. The median time from discharge to the last follow-up for patients who were still alive was 21 months. Of the 688 patients discharged to home, 414 (60.2%) died after discharge. Of the 197 patients discharged to hospice, 141 (71.6%) died, and of the 50 patients discharged to other long-term facilities, 31 (62.0%) died after discharge.

**Figure 1:**
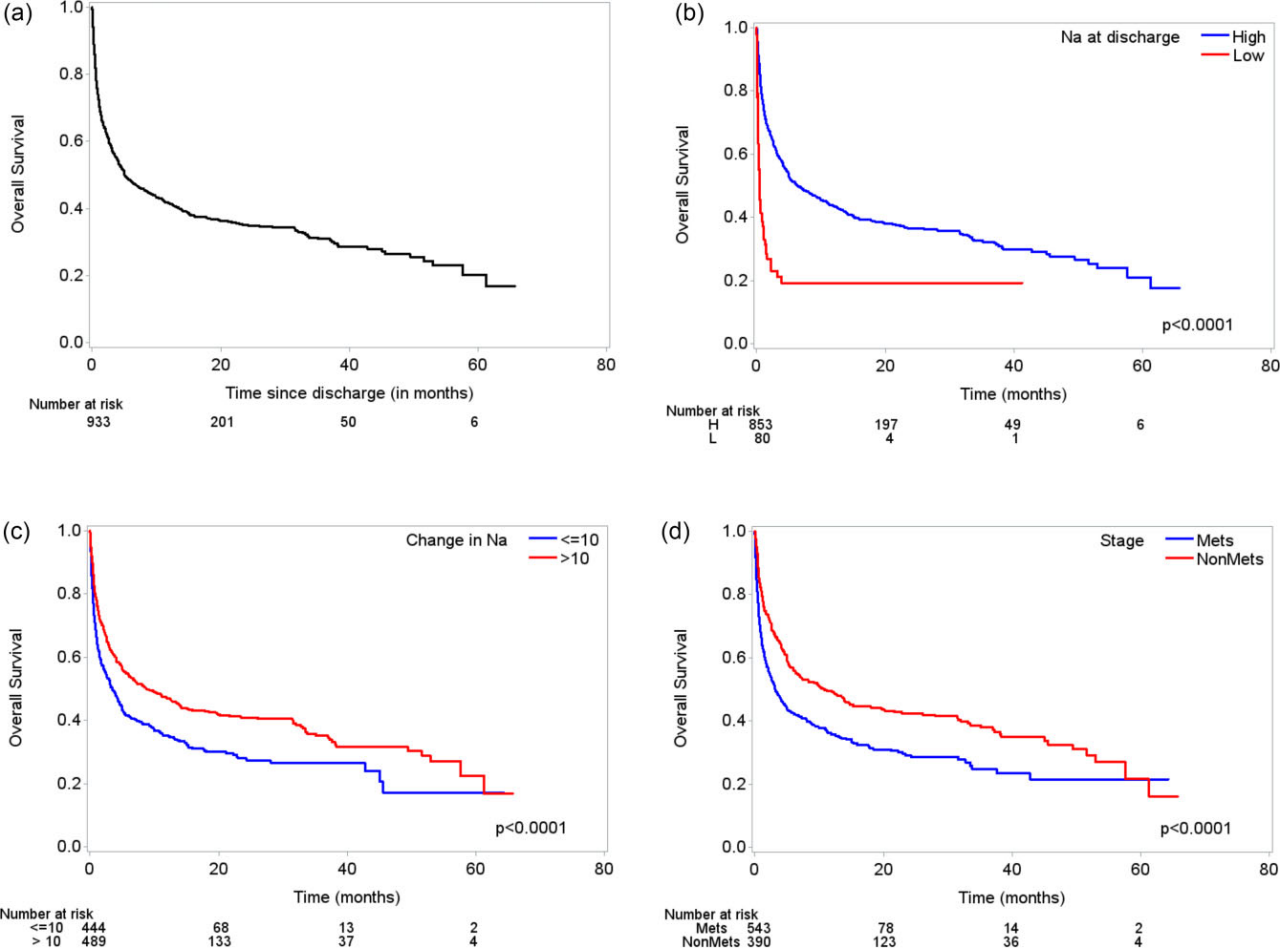
Kaplan–Meier curves for long-term survival after discharge. **(a)** Whole cohort. **(b)** By sodium level at discharge (low: ≤125, high: >125). **(c)** By change in sodium levels from admission to discharge. **(d)** By presence of metastatic disease.

In both univariate and multivariable analysis of long-term survival after hospital discharge, the following covariates were associated with poor survival: sodium levels of ≤125 mEq/l at discharge {hazard ratio [HR] 1.42 [95% confidence interval (CI) 1.02–1.98], *P* = .0382}, albumin <3.5 g/dl at admission [HR 1.48 (95% CI 1.24–1.78), *P* < .0001], metastatic cancer [HR 1.37 (95% CI 1.15–1.64), *P* = .0004], emergency admission [HR 1.20 (95% CI 1.01–1.43, *P* = .0390] and discharge to hospice care [HR 2.57 (95% 2.05–3.23), *P* < .0001) (Table 2 and Fig. 1). An increase in sodium, as well as skin cancer, was associated with better survival in both univariate and multivariable analysis [in a 1-unit sodium increase: HR 0.98 (95% CI 0.96–0.99), *P* = .0081; skin cancer: HR 0.55 (95% CI 0.39–0.75), *P* = .0002] (Table 2). Multivariable analysis performed exclusively in patients with metastatic disease showed that an increase in sodium from admission to discharge was associated with improved survival [HR 0.97 (95% CI 0.95–0.99), *P* = .0013] and patients discharged to hospice care had poorer survival than those discharged elsewhere [HR 2.77 (95% CI 2.16–3.54), *P* < .0001] (Supplementary Table S1a and Supplementary Figure S1a). When evaluating survival after discharge in the non-metastatic cohort, change in sodium was not significant in a multivariate model but sodium <125 mg/dl was associated with poorer survival [HR 2.70 (95% CI 1.42–5.15), *P* = .0025] (Supplementary Table S1b and Supplementary Figure S1b). Median long-term survival times from discharge were 6.7 months (95% CI 4.5–10.9) for those with metastatic disease and 13.6 months (95% CI 8.3–22.8) for those with non-metastatic disease.

**Table 2:** Cox regression analysis of long-term survival after hospital discharge for those discharged alive.^a^^.^

	Univariate Cox model		Multivariable Cox model	
Covariate	HR (95% CI)	*P*-value	HR (95% CI)	*P*-value
Age at discharge (years)^b^	1.00 (0.99–1.00)	.1793		
Sodium level at discharge (mEq/l)^b^	0.94 (0.92–0.96)	<.0001		
Albumin at admission (g/dl)^b^	0.57 (0.51–0.65)	<.0001		
Change in sodium level at discharge (mEq/l)^b^	0.95 (0.94–0.97)	<.0001	0.98 (0.96–0.99)	.0081
Sodium level at discharge (mEq/l)				
>125	1.00		1.00	
≤125	2.87 (2.18–3.78)	<.0001	1.42 (1.02–1.98)	.0382
Albumin at admission (g/dl)				
3.5–5.4	1.00		1.00	
<3.5	1.93 (1.63–2.27)	<.0001	1.48 (1.24–1.78)	<.0001
Age at discharge (years)				
≤65	1.00			
>65	0.94 (0.80–1.11)	.4741		
Sex				
Female	1.00			
Male	0.90 (0.77–1.06)	.2055		
Race				
White	1.00			
Black	1.24 (0.90–1.69)	.1861		
Asian	0.83 (0.63–1.10)	.1982		
Other	0.84 (0.63–1.13)	.2545		
Ethnicity				
Hispanic/Latino	1.00			
Not Hispanic/Latino	0.95 (0.77–1.18)	.6384		
Metastasis				
Non-metastatic	1.00		1.00	
Metastatic	1.49 (1.26–1.76)	<.0001	1.37 (1.15–1.64)	.0004
Admission type				
Non-emergency	1.00		1.00	
Emergency	1.18 (1.00–1.40)	.0522	1.20 (1.01–1.43)	.0390
Insurance coverage				
Commercial	1.00			
Government	0.51 (0.28–0.92)	.0260		
Medicaid/Medicare	0.82 (0.69–0.97)	.0216		
Self-pay	0.76 (0.52–1.10)	.1475		
ICU admission				
Yes	1.00			
No	1.01 (0.84–1.21)	.9465		
Cancer type				
Gastrointestinal	1.00		1.00	
Brain	0.50 (0.25–1.01)	.0523	0.74 (0.36–1.50)	.4005
Breast	0.82 (0.59–1.13)	.2251	0.96 (0.69–1.35)	.8239
Genitourinary	0.62 (0.47–0.80)	.0003	0.90 (0.68–1.18)	.4414
Gynaecologic	0.84 (0.63–1.12)	.2442	1.20 (0.89–1.61)	.2314
Head and neck	0.56 (0.40–0.78)	.0006	0.91 (0.65–1.28)	.5899
Lung	0.85 (0.66–1.11)	.2323	1.51 (1.14–2.01)	.0044
Sarcoma	0.72 (0.42–1.23)	.2271	0.90 (0.52–1.56)	.7157
Skin	0.42 (0.31–0.58)	<.0001	0.55 (0.39–0.75)	.0002
Discharge location				
Home	1.00		1.00	
Hospice	3.61 (2.96–4.39)	<.0001	2.57 (2.05–3.23)	<.0001
Another care facility	1.34 (0.93–1.93)	.1187	1.38 (0.95–2.01)	.0876
CKD				
No	1.00			
Yes	1.00 (0.81–1.24)	.9733		
Cirrhosis				
No	1.00			
Yes	1.53 (1.14–2.04)	.0040		
Diabetes				
No	1.00			
Yes	0.75 (0.24–2.33)	.6161		
Heart failure				
No	1.00			
Yes	1.04 (0.75–1.45)	.8076		
Hypertension				
No	1.00			
Yes	0.96 (0.72–1.27)	.7564		

a Includes 933 patients; 2 additional patients were discharged alive but did not follow up.

b Continuous variables calculated per 1-unit change.

In an evaluation of long-term survival after hospital discharge that excluded patients discharged to hospice, in both univariate and multivariable analyses, serum sodium ≤125 mEq/l at discharge [HR 2.17 (95% CI 1.40–3.38), *P* = .0006] and metastatic cancer [HR 1.37 (95% CI 1.13–1.67), *P* = .0014] were associated with increased mortality (Supplementary Table S2 and Supplementary Figure S3). An increase in sodium, as well as skin cancer, was associated with improved survival [increased sodium: HR 0.97 (95% CI 0.95–0.98), *P* = .0004; skin cancer: HR 0.49 (95% CI 0.34–0.70), *P* < .0001]. When the analysis was censored at 3 and 6 months, the conclusions remained the same (data not shown).

When evaluating long-term survival after discharge exclusively in patients with metastatic disease who were not discharged to hospice, we found that only an increase in sodium from admission to discharge [HR 0.96 (95% CI 0.94–0.99), *P* = .0041] was associated with improved survival. Sodium levels of ≤125 mEq/l at discharge were associated with poor survival [HR 2.74 (95% CI 1.62–4.63), *P* = .0002]. In addition, when the analysis was censored at 3 and 6 months, the conclusions remained the same (data not shown).

In both univariate and multivariable analyses of hospital survival from admission to discharge, neither sodium level at admission nor metastatic disease was associated with hospital survival. However, increasing age [HR 0.99 (95% CI 0.98–1.00), *P* = .0322], no ICU admission [HR 0.50 (95% CI 0.36–0.70), *P* < .0001] and skin cancer [HR 0.43 (95% CI 0.21–0.89), *P* = .0220] were associated with improved hospital survival (Table 3). In a multivariable analysis that included only patients with metastatic disease, cancer type and cirrhosis were associated with hospital survival (data not shown).

**Table 3:** Cox regression analysis of hospital survival from admission to discharge (*n* = 1100).

	Univariate Cox model		Multivariable Cox model	
Covariate	HR (95% CI)	*P*-value	HR (95% CI)	*P*-value
Age at admission (years)^a^	0.98 (0.97–0.99)	.0014	0.99 (0.98–1.00)	.0322
Sodium level at admission (mEq/l)^a^	0.99 (0.95–1.03)	.7066		
Albumin at admission (g/dl)^a^	0.60 (0.48–0.76)	<.0001		
Readmissions by 6 months after discharge^a^	1.03 (0.94–1.14)	.4976		
Albumin at admission (g/dl)				
3.5–5.4	1.00		1.00	
<3.5	1.92 (1.38–2.67)	.0001	1.80 (1.28–2.53)	.0008
Age at admission (years)				
≤65	1.00			
>65	0.61 (0.44–0.84)	.0031		
Sex				
Female	1.00			
Male	1.06 (0.78–1.44)	.7210		
Race				
White	1.00		1.00	
Black	1.62 (0.98–2.67)	.0575	1.53 (0.92–2.55)	.1012
Asian	0.76 (0.44–1.29)	.3050	0.51 (0.29–0.90)	.0189
Other	1.07 (0.67–1.73)	.7676	0.95 (0.58–1.56)	.8523
Ethnicity				
Hispanic/Latino	1.00			
Not Hispanic/Latino	1.15 (0.76–1.73)	.5025		
Metastasis				
Non-metastatic	1.00			
Metastatic	1.14 (0.83–1.56)	.4058		
Admission type				
Non-emergency	1.00			
Emergency	1.36 (0.97–1.90)	.0707		
Insurance coverage				
Commercial	1.00			
Government	0.72 (0.29–1.76)	.4706		
Medicaid/Medicare	0.56 (0.38–0.81)	.0025		
Self-pay	0.61 (0.31–1.21)	.1566		
ICU admission				
Yes	1.00		1.00	
No	0.59 (0.43–0.81)	.0012	0.50 (0.36–0.70)	<.0001
Cancer type				
Gastrointestinal	1.00		1.00	
Brain	0.38 (0.05–2.75)	.3383	0.45 (0.06–3.30)	.4322
Breast	1.64 (0.92–2.92)	.0907	1.99 (1.11–3.57)	.0211
Genitourinary	0.44 (0.24–0.81)	.0081	0.43 (0.23–0.81)	.0093
Gynaecologic	0.75 (0.37–1.50)	.4174	0.75 (0.36–1.57)	.4444
Head and neck	0.48 (0.22–1.05)	.0677	0.45 (0.20–1.02)	.0545
Lung	1.16 (0.75–1.79)	.4935	1.30 (0.83–2.03)	.2564
Sarcoma	1.45 (0.78–2.68)	.2398	1.43 (0.76–2.69)	.2697
Skin	0.40 (0.20–0.80)	.0097	0.43 (0.21–0.89)	.0220
CKD				
No	1.00			
Yes	0.86 (0.57–1.32)	.4978		
Cirrhosis				
No	1.00			
Yes	1.34 (0.81–2.22)	.2509		
Diabetes				
No	1.00			
Yes	2.37 (0.88–6.42)	.0883		
Heart failure				
No	1.00			
Yes	1.29 (0.79–2.11)	.3082		
Hypertension				
No	1.00			
Yes	1.31 (0.81–2.11)	.2757		

a Continuous variables calculated per 1-unit change.

**Figure 2:**
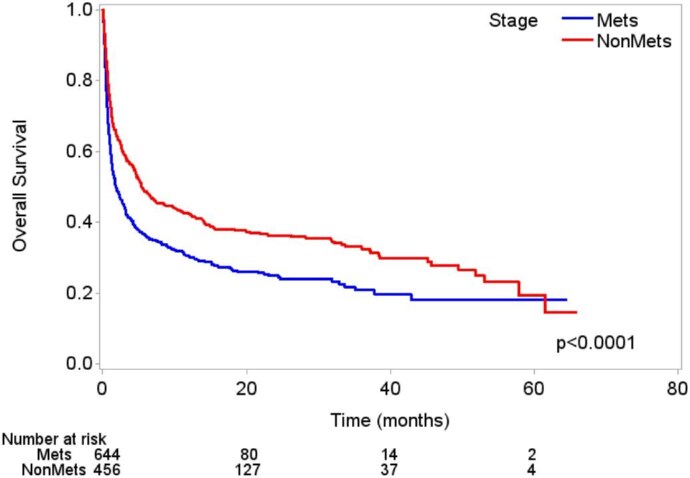
Kaplan–Meier curves for overall survival from hospital admission to last follow-up for all patients.

A final analysis was performed to evaluate overall survival from admission to last follow-up, and in both univariate and multivariable analyses, patients admitted through the emergency room [HR 1.23 (95% CI 1.05–1.43), *P* = .0087] had poorer overall survival than those admitted directly to the floor. Conversely, patients admitted without requiring an ICU stay [HR 0.79 (95% CI 0.67–0.92), *P* = .0030] or with skin, head and neck or genitourinary cancer all had better overall survival (*P* < .05; Table 4). Patients with metastatic disease had poorer overall survival at last follow-up in the univariate and multivariable models [HR 1.30 (95% CI 1.11–1.52), *P* = .0010; Table 4 and Fig. 2).

**Table 4:** Cox regression analysis of overall survival from hospital admission to last follow-up (*n* = 1100).

	Univariate Cox model		Multivariable Cox model	
Covariate	HR (95% CI)	*P*	HR (95% CI)	*P*
Age at admission (years)^a^	0.99 (0.98–1.00)	.0007		
Sodium level at admission (mEq/l)^a^	1.01 (0.99–1.03)	.3505		
Albumin at admission (g/dl)^a^	0.55 (0.50–0.61)	<.0001		
Readmissions by 6 months after discharge^a^	1.04 (0.99–1.10)	.1032		
Albumin at admission (g/dl)				
3.5–5.4	1.00		1.00	
<3.5	2.02 (1.75–2.34)	<.0001	1.92 (1.65–2.25)	<.0001
Age at admission (years)				
≤65	1.00			
>65	0.84 (0.73–0.98)	.0220		
Sex				
Female	1.00			
Male	0.97 (0.84–1.12)	.6755		
Race				
White	1.00		1.00	
Black	1.33 (1.02–1.73)	.0361	1.18 (0.90–1.55)	.2228
Asian	0.85 (0.67–1.10)	.2168	0.73 (0.57–0.94)	.0154
Other	0.92 (0.72–1.19)	.5357	0.80 (0.62–1.04)	.0906
Ethnicity				
Hispanic/Latino	1.00			
Not Hispanic/Latino	0.98 (0.81–1.19)	.8602		
Metastasis				
Non-metastatic	1.00		1.00	
Metastatic	1.42 (1.22–1.64)	<.0001	1.30 (1.11–1.52)	.0010
Admission type				
Non-emergency	1.00		1.00	
Emergency	1.24 (1.07–1.44)	.0051	1.23 (1.05–1.43)	.0087
Insurance coverage				
Commercial	1.00		1.00	
Government	0.56 (0.34–0.93)	.0240	0.58 (0.35–0.98)	.0398
Medicaid/Medicare	0.75 (0.64–0.87)	.0003	0.87 (0.74–1.02)	.0827
Self-pay	0.75 (0.54–1.05)	.0925	0.76 (0.55–1.06)	.1094
Intensive care unit admission				
Yes	1.00		1.00	
No	0.77 (0.66–0.89)	.0006	0.79 (0.67–0.92)	.0030
Cancer type				
Gastrointestinal	1.00		1.00	
Brain	0.46 (0.24–0.90)	.0229	0.67 (0.34–1.31)	.2372
Breast	0.88 (0.66–1.17)	.3765	0.96 (0.72–1.28)	.7670
Genitourinary	0.58 (0.46–0.74)	<.0001	0.65 (0.51–0.84)	.0007
Gynaecologic	0.79 (0.61–1.03)	.0809	0.97 (0.74–1.27)	.8075
Head and neck	0.54 (0.40–0.73)	<.0001	0.70 (0.51–0.95)	.0237
Lung	0.90 (0.72–1.12)	.3397	1.16 (0.92–1.47)	.2147
Sarcoma	0.98 (0.65–1.46)	.9021	0.97 (0.64–1.46)	.8731
Skin	0.42 (0.32–0.57)	<.0001	0.46 (0.34–0.62)	<.0001
CKD				
No	1.00			
Yes	0.99 (0.82–1.19)	.8817		
Cirrhosis				
No	1.00			
Yes	1.53 (1.19–1.97)	.0008		
Diabetes				
No	1.00			
Yes	1.24 (0.59–2.61)	.5682		
Heart failure				
No	1.00			
Yes	1.17 (0.89–1.54)	.2460		
Hypertension				
No	1.00			
Yes	1.01 (0.79–1.29)	.9537		

a Continuous variables calculated per 1-unit change.

In multivariable analysis of overall survival from admission to last follow-up only in patients with metastatic disease, patients admitted through the emergency room had poorer overall survival [HR 1.62 (95% CI 1.33–1.97), *P* < .0001] and patients with genitourinary cancer or skin cancer had better overall survival (*P* < .001). The median overall survival times (admission to last follow-up) for patients with non-metastatic disease were longer than those for patients with metastatic disease [5.4 months (95% CI 4.5–8.4) compared with 1.9 months (95% CI 1.4–2.6)].

## DISCUSSION

Hyponatraemia is a common complication observed in cancer patients and has been consistently associated with adverse outcomes, including prolonged hospitalization, increased healthcare costs and higher mortality rates [12, 14]. However, understanding the role of hyponatraemia in patients with advanced cancer has been limited by conflicting evidence [11, 15, 19, 21]. The current retrospective study aimed to investigate the impact of hyponatraemia on overall survival among hospitalized patients with advanced cancer. We found that in patients with metastatic cancer, being discharged with sodium <125 mEq/l was associated with a decrease in overall survival. The concern in advanced cancer patients is whether the hyponatraemia is the cause of death or a prognostic indicator of advanced disease.

These findings bring awareness to important aspects concerning the relationship between sodium levels, cancer stage, discharge disposition and overall survival. When evaluating hospital survival from admission to discharge, sodium level at admission was not an independent predictor of overall survival, but admission through the emergency room or ICU were independently associated with poor survival. We speculate that the lack of correlation between sodium level on admission and inpatient survival was related to the impact of an acute illness or other disease associated factors on survival, in contrast to patients with less severe hyponatraemia [6]. In addition, in this study we only included patients with severe hyponatraemia (<125 mEq/l), where the mortality rate might not be directly correlated with sodium level, in contrast to patients with less severe hyponatraemia [6, 22].

Nonetheless, we observed an improvement in patient survival, both with metastatic and non-metastatic cancer, by correcting hyponatraemia to at least 125 mEq/l at the time of discharge. Interestingly, an increase in sodium level of 1 mEq/l was associated with improved survival in the metastatic cancer subgroup but not in the non-metastatic subgroup. This might be related to the variability of the hyponatraemia correction rate, which can impact of mortality. In a multicentre observational study that included 3274 patients with severe hyponatraemia on admission (<120 mEq/l), 57% had history of malignancy or metastatic disease, and correcting serum sodium to <6 mEq/l per 24 h was associated with a higher mortality rate compared with >6 mEq/l per 24 h [22].

Several attempts have been made to evaluate patients with advanced cancer and hyponatraemia. Yoon *et al.* [15] evaluated cancer patients in a palliative care unit, where 85.6% had metastatic cancer and 9.4% of them had a serum sodium level <125 mEq/l. Multivariable analysis indicated that low sodium was strongly associated with poor survival [HR 1.75 (95% CI 1.29–2.38), *P* < .001]. In the HYPNOSIS study of 1025 patients admitted to a cancer centre with metastatic solid tumours, hyponatraemia was also associated with poorer overall survival independent of other variables [HR 1.66 (95% CI 1.38–2.01), *P* < .001], and overall survival outcomes were worse with sodium <125 mEq/l [11]. However, Ferraz Goncalves *et al.* [19] evaluated 300 patients admitted to a palliative care department, where 4% had a serum sodium level <125 mEq/l, and in that study, hyponatraemia was not associated with overall survival; only Eastern Cooperative Oncology Group status was associated with overall survival (*P* < .001).

Our study also highlighted the impact of cancer stage on discharge disposition and subsequent survival. Patients with metastatic cancer faced higher mortality rates across various discharge destinations, including home and hospice, compared with those with non-metastatic disease. Although improved sodium levels on discharge was associated with better survival, we noted that median overall survival after discharge in patients with metastatic cancer was 6.7 months, compared with 13.6 months in those with non-metastatic disease, and more readmissions occurred in patients with metastatic disease compared with those with non-metastatic disease (48% had more than one readmission, compared with 40%). This emphasizes the critical need for tailored care strategies and early palliative interventions for patients with advanced cancer to improve their quality of life and prognosis.

Many patients with advanced cancer also have cachexia syndrome, affecting their fluid and calorie intake and leading to failure to thrive [23]. In addition, multiple studies have indicated that hyponatraemia is an independent prognostic indicator of poor prognosis among patients with advanced cancer, especially in those with lung cancer [12, 13, 24]. Patients with advanced solid tumours are at risk of developing malignant ascites, which require frequent paracentesis or placement of an intraperitoneal catheter [25]. Almost all of these patients are at risk of significant electrolyte imbalance, especially hyponatraemia. However, given that these measures are essential to improving quality of life, treating hyponatraemia in this population must be weighed carefully [25]. Rather than focusing on hyponatraemia, it is prudent that clinicians have an honest and realistic discussion with patients and their families about the overall prognosis and understand their wishes.

The current study also addressed the economic implications of hyponatraemia in cancer patients, revealing higher total charges associated with hospital admissions for patients with metastatic disease compared with those with non-metastatic cancer. This underscores the significant healthcare utilization and financial burden associated with advanced cancer complicated by hyponatraemia.

The current study, being a single-centre retrospective study, has some limitations, such as the lack of comorbidity scores and further data about the causes of hyponatraemia and interventions taken. We included only patients who had a nephrology consultation to ensure that the hyponatraemia was addressed during the patient's admission, and we collected sodium values at both admission and discharge. Interestingly, when we evaluated survival from hospital admission to discharge, increasing age was an independent predictor of improved survival. Although this was also demonstrated in another recent study, the finding needs to be further validated [22]. A possible explanation is that the younger patients in our cohort could have been sicker than the older ones. Prospective studies would be needed to compare severe hyponatraemia in patients with advanced solid cancer with that of patients with non-metastatic disease, including propensity score matching to evaluate more accurately the associated mortality, morbidity and overall cost of hyponatraemia in these patients.

The findings of the current study have several clinical implications. First, our findings emphasize the importance of closely monitoring and managing sodium levels in cancer patients, particularly those with advanced disease, to potentially improve survival outcomes. Second, there is a need for comprehensive, multidisciplinary care approaches that integrate early palliative care interventions to optimize patient outcomes and quality of life. Finally, the economic burden associated with hyponatraemia underscores the importance of cost-effective interventions and resource allocation strategies in cancer care.

## CONCLUSIONS

Our study demonstrated that upon discharge of metastatic cancer patients, improved sodium in patients with severe hyponatraemia (<125 mEq/l) on admission is associated with improved survival. However, metastatic disease itself is an independent predictor of poor survival. We do recommend correcting sodium in advanced cancer patients, but also to be aware that hyponatraemia in itself could be an indicator of progressive disease. A multidisciplinary approach is needed to design a prognostic tool for this population to help guide management and early palliative care consultations, thereby improving end-of-life care.

## Supplementary Material

sfaf023_Supplemental_File

## Data Availability

The data underlying this article are available in the article and in its online supplementary material.
