# Supplementary material for: Correcting hyponatraemia is associated with improved survival in hyponatraemic metastatic cancer patients
Source: Clin Kidney J. 2025 Jan 24;18(3):sfaf023. doi: 10.1093/ckj/sfaf023 (PMC11883221; doi:10.1093/ckj/sfaf023)

**Supplementary Tables and Figures**

**Table S1a.** Cox regression analysis of long-term survival after hospital discharge for those discharged alive who had metastatic cancer (n = 644).

| **Covariate** | **Univariate Cox model** | | **Multivariable Cox model** | |
| --- | --- | --- | --- | --- |
|  | **HR (95% CI)** | ***P*** | **HR (95% CI)** | ***P*** |
| Age at discharge, years^a^ | 0.99 (0.99-1.00) | 0.1852 |  |  |
| Sodium level at discharge, mEq/L^a^ | 0.94 (0.92-0.96) | <0.0001 |  |  |
| Albumin at admission^a^ | 0.59 (0.50-0.70) | <0.0001 |  |  |
| Change in sodium level at discharge, mEq/L^a^ | 0.95 (0.93-0.97) | <0.0001 | 0.97 (0.95-0.99) | 0.0013 |
| Sodium level at discharge |  |  |  |  |
| >125 mEq/L | 1.00 |  |  |  |
| ≤125 mEq/L | 2.53 (1.84-3.48) | <0.0001 |  |  |
| Albumin at admission |  |  |  |  |
| 3.5-5.4 | 1.00 |  | 1.00 |  |
| <3.5 | 1.74 (1.40-2.15) | <0.0001 | 1.37 (1.09-1.71) | 0.0064 |
| Age at discharge |  |  |  |  |
| ≤65 years | 1.00 |  |  |  |
| >65 years | 0.87 (0.70-1.07) | 0.1951 |  |  |
| Sex |  |  |  |  |
| Female | 1.00 |  |  |  |
| Male | 0.97 (0.79-1.19) | 0.7607 |  |  |
| Race |  |  |  |  |
| White | 1.00 |  |  |  |
| Black | 1.17 (0.78-1.75) | 0.4512 |  |  |
| Asian | 0.97 (0.69-1.37) | 0.8657 |  |  |
| Other | 0.94 (0.66-1.34) | 0.7322 |  |  |
| Ethnicity |  |  |  |  |
| Hispanic/Latino | 1.00 |  |  |  |
| Not Hispanic/Latino | 0.88 (0.68-1.15) | 0.3515 |  |  |
| Admission type |  |  |  |  |
| Non-emergency | 1.00 |  | 1.00 |  |
| Emergency | 1.30 (1.05-1.61) | 0.0183 | 1.30 (1.04-1.62) | 0.0207 |
| Insurance coverage |  |  |  |  |
| Commercial | 1.00 |  |  |  |
| Government | 0.58 (0.26-1.31) | 0.1931 |  |  |
| Medicaid/Medicare | 0.79 (0.63-1.00) | 0.0469 |  |  |
| Self-pay | 0.72 (0.45-1.17) | 0.1881 |  |  |
| Intensive care unit admission |  |  |  |  |
| Yes | 1.00 |  |  |  |
| No | 1.05 (0.83-1.33) | 0.6818 |  |  |
| Cancer type |  |  |  |  |
| Gastrointestinal | 1.00 |  |  |  |
| Brain | 0.59 (0.22-1.59) | 0.2948 |  |  |
| Breast | 0.91 (0.61-1.35) | 0.6314 |  |  |
| Genitourinary | 0.54 (0.39-0.77) | 0.0005 |  |  |
| Gynecologic | 0.76 (0.52-1.10) | 0.1448 |  |  |
| Head and neck | 0.72 (0.46-1.13) | 0.1560 |  |  |
| Lung | 0.80 (0.51-1.26) | 0.3359 |  |  |
| Sarcoma | 0.71 (0.39-1.32) | 0.2805 |  |  |
| Skin | 0.46 (0.32-0.66) | <0.0001 |  |  |
| Discharge location |  |  |  |  |
| Home | 1.00 |  | 1.00 |  |
| Hospice | 3.38 (2.68-4.27) | <0.0001 | 2.77 (2.16-3.54) | <0.0001 |
| Another care facility | 0.91 (0.51-1.63) | 0.7506 | 1.03 (0.57-1.86) | 0.9265 |
| CKD |  |  |  |  |
| No | 1.00 |  |  |  |
| Yes | 0.92 (0.69-1.23) | 0.5765 |  |  |
| Cirrhosis |  |  |  |  |
| No | 1.00 |  |  |  |
| Yes | 1.41 (1.00-1.99) | 0.0525 |  |  |
| Diabetes |  |  |  |  |
| No | 1.00 |  |  |  |
| Yes | 0.29 (0.04-2.07) | 0.2167 |  |  |
| Heart failure |  |  |  |  |
| No | 1.00 |  |  |  |
| Yes | 0.70 (0.43-1.16) | 0.1711 |  |  |
| Hypertension |  |  |  |  |
| No | 1.00 |  |  |  |
| Yes | 1.04 (0.70-1.54) | 0.8344 |  |  |

^a^Continuous variables calculated per 1-unit change.

Table S1b: Cox regression analysis of long-term survival after hospital discharge for those discharged alive who had non-metastatic cancer (n = 391).

| **Covariate** | **Univariate Cox model** | | **Multivariable Cox model** | |
| --- | --- | --- | --- | --- |
|  | **HR (95% CI)** | ***P*** | **HR (95% CI)** | ***P*** |
| Age at discharge, years^a^ | 1.00 (0.99-1.01) | 0.6171 |  |  |
| Sodium level at discharge, mEq/L^a^ | 0.96 (0.93-0.99) | 0.0200 |  |  |
| Albumin at admission^a^ | 0.60 (0.50-0.72) | <0.0001 |  |  |
| Change in sodium level at discharge, mEq/L^a^ | 0.97 (0.94-0.99) | 0.0154 |  |  |
| Sodium level at discharge |  |  |  |  |
| >125 mEq/L | 1.00 |  | 1.00 |  |
| ≤125 mEq/L | 2.93 (1.67-5.14) | 0.0002 | 2.70 (1.42-5.15) | 0.0025 |
| Albumin at admission |  |  |  |  |
| 3.5-5.4 | 1.00 |  | 1.00 |  |
| <3.5 | 1.94 (1.49-2.53) | <0.0001 | 1.73 (1.29-2.32) | 0.0002 |
| Age at discharge |  |  |  |  |
| ≤65 years | 1.00 |  |  |  |
| >65 years | 1.13 (0.87-1.46) | 0.3576 |  |  |
| Sex |  |  |  |  |
| Female | 1.00 |  |  |  |
| Male | 0.83 (0.64-1.07) | 0.1510 |  |  |
| Race |  |  |  |  |
| White | 1.00 |  | 1.00 |  |
| Black | 1.32 (0.80-2.17) | 0.2753 | 1.37 (0.82-2.27) | 0.2283 |
| Asian | 0.62 (0.38-1.02) | 0.0621 | 0.53 (0.32-0.87) | 0.0131 |
| Other | 0.67 (0.40-1.12) | 0.1263 | 0.56 (0.33-0.95) | 0.0322 |
| Ethnicity |  |  |  |  |
| Hispanic/Latino | 1.00 |  |  |  |
| Not Hispanic/Latino | 1.12 (0.77-1.62) | 0.5465 |  |  |
| Admission type |  |  |  |  |
| Non-emergency | 1.00 |  | 1.00 |  |
| Emergency | 1.05 (0.81-1.38) | 0.6996 | 1.30 (1.04-1.62) | 0.0207 |
| Insurance coverage |  |  |  |  |
| Commercial | 1.00 |  |  |  |
| Government | 0.50 (0.20-1.23) | 0.1305 |  |  |
| Medicaid/Medicare | 0.95 (0.72-1.24) | 0.6914 |  |  |
| Self-pay | 0.83 (0.46-1.51) | 0.5484 |  |  |
| Intensive care unit admission |  |  |  |  |
| Yes | 1.00 |  |  |  |
| No | 0.99 (0.73-1.33) | 0.9343 |  |  |
| Cancer type |  |  |  |  |
| Gastrointestinal | 1.00 |  | 1.00 |  |
| Brain | 0.53 (0.19-1.46) | 0.2206 | 0.74 (0.27-2.05) | 0.5635 |
| Breast | 0.72 (0.40-1.31) | 0.2830 | 0.49 (0.26-0.96) | 0.0373 |
| Genitourinary | 0.83 (0.54-1.27) | 0.3796 | 0.98 (0.64-1.53) | 0.9441 |
| Gynecologic | 1.08 (0.68-1.70) | 0.7537 | 1.20 (0.75-1.91) | 0.4454 |
| Head and neck | 0.55 (0.33-0.91) | 0.0201 | 0.66 (0.39-1.09) | 0.1061 |
| Lung | 1.15 (0.80-1.64) | 0.4498 | 1.52 (1.04-2.23) | 0.0320 |
| Sarcoma | 0.63 (0.20-2.01) | 0.4356 | 0.73 (0.22-2.35) | 0.5941 |
| Skin | 0.35 (0.19-0.65) | 0.0009 | 0.40 (0.21-0.76) | 0.0048 |
| Discharge location |  |  |  |  |
| Home | 1.00 |  | 1.00 |  |
| Hospice | 3.11 (2.08-4.65) | <0.0001 | 2.56 (1.61-4.05) | <0.0001 |
| Another care facility | 1.97 (1.22-3.16) | 0.0052 | 2.14 (1.30-3.52) | 0.0029 |
| CKD |  |  |  |  |
| No | 1.00 |  |  |  |
| Yes | 1.20 (0.88-1.63) | 0.2527 |  |  |
| Cirrhosis |  |  |  |  |
| No | 1.00 |  |  |  |
| Yes | 1.60 (0.95-2.71) | 0.0775 |  |  |
| Diabetes |  |  |  |  |
| No | 1.00 |  |  |  |
| Yes | 2.72 (0.67-11.01) | 0.1605 |  |  |
| Heart failure |  |  |  |  |
| No | 1.00 |  | 1.00 |  |
| Yes | 1.70 (1.09-2.64) | 0.0187 | 1.88 (1.20-2.95) | 0.0058 |
| Hypertension |  |  |  |  |
| No | 1.00 |  |  |  |
| Yes | 0.93 (0.61-1.43) | 0.7500 |  |  |

**Table S2.** Cox regression analysis of long-term survival after hospital discharge for those not discharged to hospice (n = 738).

| **Covariate** | **Univariate Cox model** | | **Multivariate Cox model** | |
| --- | --- | --- | --- | --- |
|  | **HR (95% CI)** | ***P*** | **HR (95% CI)** | ***P*** |
| Age at discharge, years^a^ | 0.99 (0.99-1.00) | 0.2137 |  |  |
| Sodium level at discharge, mEq/L^a^ | 0.94 (0.92-0.96) | <0.0001 |  |  |
| Albumin at admission^a^ | 0.59 (0.51-0.67) | <0.0001 |  |  |
| Change in sodium level at discharge, mEq/L^a^ | 0.96 (0.94-0.97) | <0.0001 | 0.97 (0.95-0.98) | 0.0004 |
| Sodium level at discharge |  |  |  |  |
| >125 mEq/L | 1.00 |  | 1.00 |  |
| ≤125 mEq/L | 3.07 (2.03-4.65) | <0.0001 | 2.17 (1.40-3.38) | 0.0006 |
| Albumin at admission |  |  |  |  |
| 3.5-5.4 | 1.00 |  |  |  |
| <3.5 | 1.81 (1.50-2.19) | <0.0001 |  |  |
| Age at discharge |  |  |  |  |
| ≤65 years | 1.00 |  |  |  |
| >65 years | 0.95 (0.79-1.15) | 0.6154 |  |  |
| Sex |  |  |  |  |
| Female | 1.00 |  |  |  |
| Male | 0.87 (0.72-1.04) | 0.1286 |  |  |
| Race |  |  |  |  |
| White | 1.00 |  | 1.00 |  |
| Black | 1.45 (1.02-2.06) | 0.0366 | 1.44 (1.01-2.06) | 0.0418 |
| Asian | 0.79 (0.57-1.11) | 0.1774 | 0.68 (0.48-0.96) | 0.0274 |
| Other | 0.89 (0.63-1.26) | 0.5222 | 0.87 (0.61-1.23) | 0.4279 |
| Ethnicity |  |  |  |  |
| Hispanic/Latino | 1.00 |  |  |  |
| Not Hispanic/Latino | 0.90 (0.70-1.16) | 0.4132 |  |  |
| Metastasis |  |  |  |  |
| Non-metastatic | 1.00 |  | 1.00 |  |
| Metastatic | 1.29 (1.07-1.56) | 0.0077 | 1.37 (1.13-1.67) | 0.0014 |
| Admission type |  |  |  |  |
| Non-emergency | 1.00 |  |  |  |
| Emergency | 1.09 (0.90-1.32) | 0.3644 |  |  |
| Insurance coverage |  |  |  |  |
| Commercial | 1.00 |  |  |  |
| Government | 0.49 (0.25-0.96) | 0.0363 |  |  |
| Medicaid/Medicare | 0.80 (0.66-0.98) | 0.0280 |  |  |
| Self-pay | 0.71 (0.46-1.11) | 0.1335 |  |  |
| Intensive care unit admission |  |  |  |  |
| Yes | 1.00 |  |  |  |
| No | 1.11 (0.89-1.38) | 0.3477 |  |  |
| Cancer type |  |  |  |  |
| Gastrointestinal | 1.00 |  | 1.00 |  |
| Brain | 0.59 (0.28-1.26) | 0.1754 | 0.70 (0.32-1.49) | 0.3502 |
| Breast | 0.79 (0.53-1.17) | 0.2444 | 0.79 (0.53-1.18) | 0.2426 |
| Genitourinary | 0.65 (0.48-0.88) | 0.0054 | 0.67 (0.49-0.90) | 0.0084 |
| Gynecologic | 0.92 (0.66-1.29) | 0.6413 | 0.97 (0.70-1.36) | 0.8741 |
| Head and neck | 0.57 (0.39-0.83) | 0.0032 | 0.63 (0.43-0.91) | 0.0151 |
| Lung | 1.01 (0.76-1.34) | 0.9274 | 1.18 (0.88-1.58) | 0.2614 |
| Sarcoma | 0.60 (0.30-1.17) | 0.1344 | 0.53 (0.26-1.09) | 0.0824 |
| Skin | 0.46 (0.32-0.65) | <0.0001 | 0.49 (0.34-0.70) | <0.0001 |
| Discharge location |  |  |  |  |
| Home | 1.00 |  | 1.00 |  |
| Another care facility | 1.37 (0.95-1.98) | 0.0904 | 1.68 (1.15-2.45) | 0.0072 |
| CKD |  |  |  |  |
| No | 1.00 |  |  |  |
| Yes | 0.96 (0.76-1.22) | 0.7510 |  |  |
| Cirrhosis |  |  |  |  |
| No | 1.00 |  |  |  |
| Yes | 1.21 (0.83-1.78) | 0.3209 |  |  |
| Diabetes |  |  |  |  |
| No | 1.00 |  |  |  |
| Yes | 0.92 (0.30-2.87) | 0.8873 |  |  |
| Heart failure |  |  |  |  |
| No | 1.00 |  |  |  |
| Yes | 1.13 (0.79-1.63) | 0.5047 |  |  |
| Hypertension |  |  |  |  |
| No | 1.00 |  |  |  |
| Yes | 0.95 (0.69-1.33) | 0.7802 |  |  |

^a^Continuous variables calculated per 1-unit change.

**Figure S1. Kaplan-Meier curves for long-term survival after hospital discharge for patients with metastatic cancer.** **a.** All patients with metastatic cancer. **b.** By sodium level at discharge (low: ≤125, high: >125). **c.** By change in sodium levels from admission to discharge.

**a**


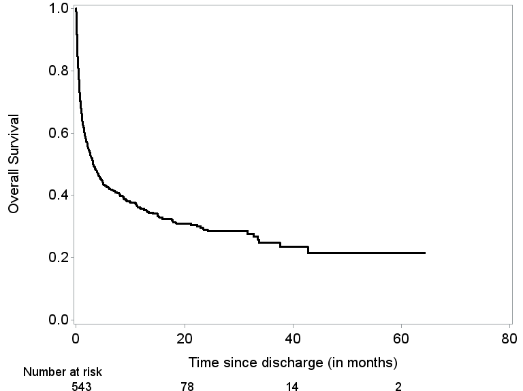


**b**


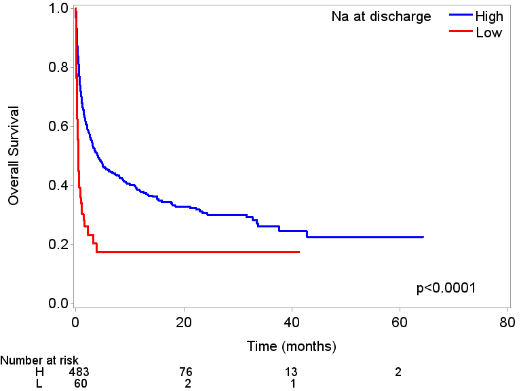


**c**


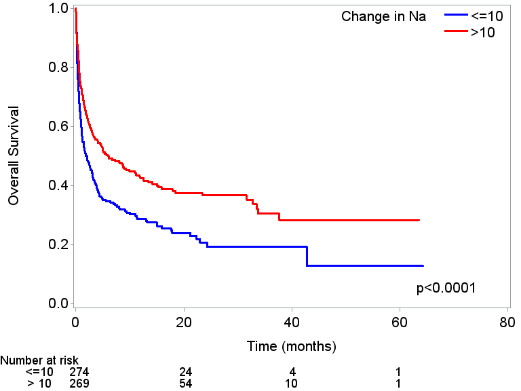


**Figure S2:**


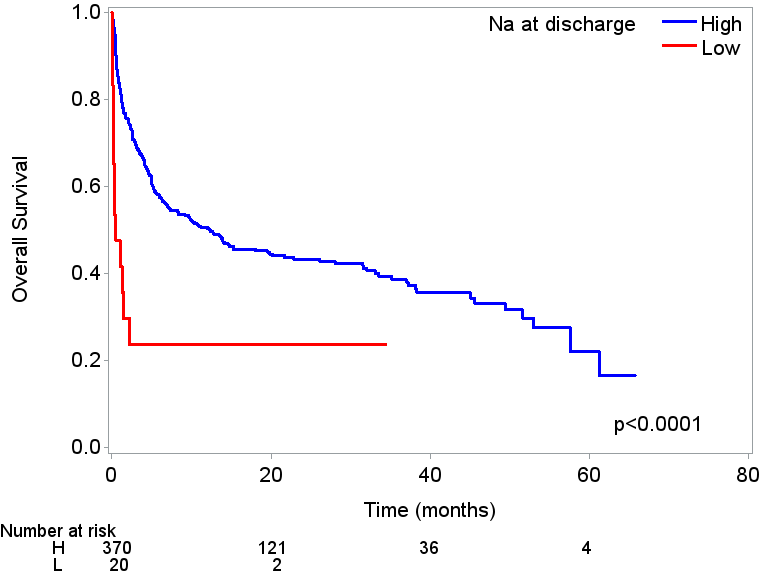


**Figure S2a:Kaplan-Meier survival estimates since discharge in non-metastatic patients by Na at discharge**

**Low: Na<=125**

**High: Na>125**


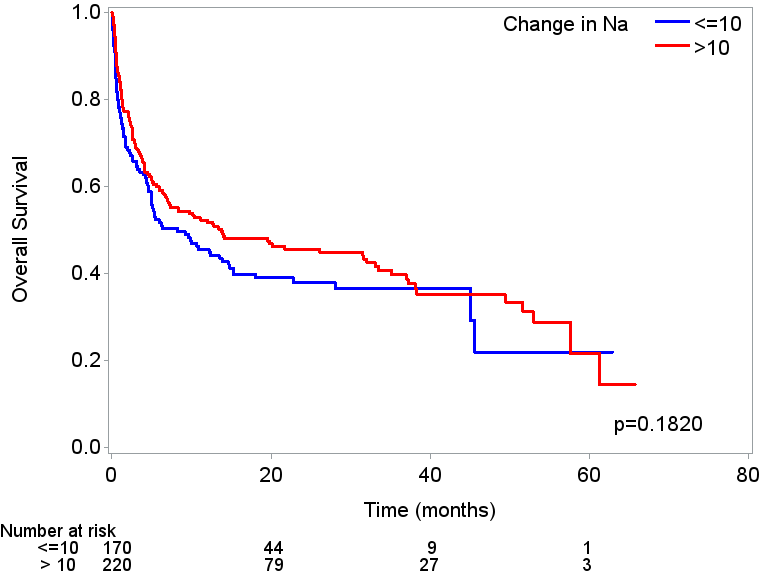


**Figure S2b:**

**Kaplan-Meier survival estimates since discharge by change in Na**

**Figure S3. Kaplan-Meier curves for long-term survival after hospital discharge, excluding those discharged to hospice.** **a.** All patients not discharged to hospice. **b.** By sodium level at discharge. **c.** By presence of metastasis.

**a**


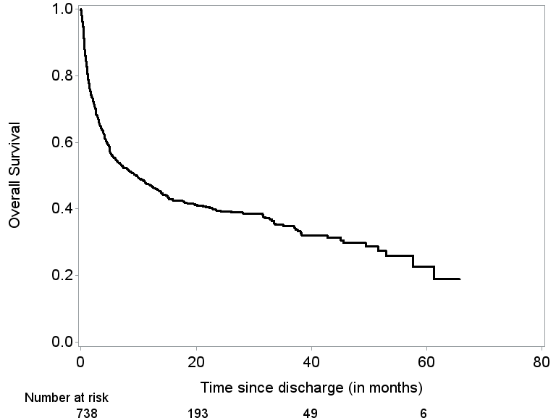


**b**


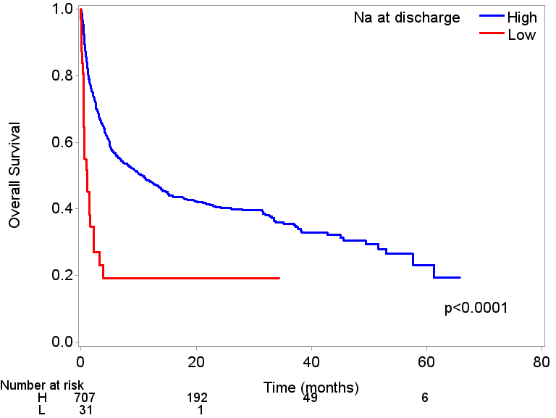


**c**


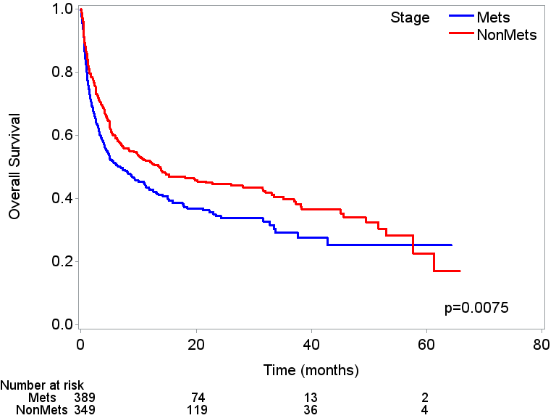

Supplement: sfaf023_Supplemental_File [file sfaf023_supplemental_file.docx]
